# A Cross-Sectional Survey on the Management of Medication Adherence Among Healthcare Professionals in Saudi Arabia

**DOI:** 10.3390/healthcare13030347

**Published:** 2025-02-06

**Authors:** Wael Y. Khawagi, Fahad H. Baali, Norah M. Alnefaie, Shatha A. Albishi, Alla H. Al-swat, Dinan A. Alshahrani, Ragad A. Alshemaimri, Abdullah A. Alshehri

**Affiliations:** 1 Department of Clinical Pharmacy, College of Pharmacy, Taif University, Taif 21944, Saudi Arabia; f.baali@tu.edu.sa (F.H.B.); a.aalshehri@tu.edu.sa (A.A.A.); 2 College of Pharmacy, Taif University, Taif 21944, Saudi Arabia

**Keywords:** medication adherence, healthcare professionals, Saudi Arabia, patient education, adherence assessment methods

## Abstract

Background/Objectives: Medication adherence is essential for effective healthcare, significantly influencing treatment success and overall health outcomes. However, there is limited understanding of how healthcare professionals in Saudi Arabia manage and support medication adherence. This study aims to examine physicians’ and pharmacists’ approaches to managing medication adherence in Saudi Arabia by examining the methods used for adherence assessment, interventions to enhance adherence, and the challenges faced. Methods: A cross-sectional study was conducted over nine months using a self-administered online questionnaire. The study targeted physicians and pharmacists actively working in Saudi Arabia. The questionnaire was distributed through professional networks to ensure a broad and representative sample. Results: A total of 397 healthcare professionals met the inclusion criteria, comprising 81.1% pharmacists and 18.9% physicians. Direct patient inquiry was the most common assessment method, frequently or always used by 81.3% of physicians and 57.1% of pharmacists. Treatment response evaluation was similarly frequent (89.3% of physicians and 56.2% of pharmacists). Standardized tools, such as the Morisky Medication Adherence Scale, were underutilized (14.7%). Adherence-enhancing interventions focused on patient education, and their use was reported by 89.3% of physicians and 74.2% of pharmacists as frequent or always. Written information was more commonly used by pharmacists (65.8%) than physicians (45.3%). Barriers included excessive workloads and short consultation times (59.9%), absence of effective systems for tracking adherence (51.9%), lack of reliable tools for assessing adherence (48.9%), and insufficient training in behavioral interventions (48.1%). Conclusions: This study reveals significant differences in medication adherence management practices between physicians and pharmacists in Saudi Arabia, emphasizing their distinct roles. Key barriers, including excessive workload, limited consultation time, and inadequate tracking systems, hinder the adoption of evidence-based tools. Tailored interventions, enhanced interprofessional collaboration, and systemic support are essential to address these challenges and improve adherence management, ultimately enhancing patient outcomes.

## 1. Introduction

Medication adherence is a critical determinant of treatment efficacy, particularly in managing chronic conditions such as hypertension, diabetes, and cardiovascular diseases, which are prevalent in Saudi Arabia [1,2]. Despite its importance, approximately 50% of patients with chronic illnesses in the country fail to adhere to their prescribed medication regimens, leading to worsened disease outcomes, increased mortality rates, and elevated healthcare costs [3,4]. Studies indicate that patients who adhere to their treatments experience lower mortality and fewer hospitalizations, underscoring the need to improve adherence rates to enhance health outcomes and reduce the burden of chronic diseases [5,6].

Healthcare professionals play a pivotal role in promoting medication adherence. Despite their efforts, they face numerous challenges, including the complexities of patient behavior, limited consultation time, and selecting appropriate assessment methods. The research suggests that healthcare professionals often underestimate the prevalence of non-adherence, highlighting a significant gap in their ability to address this issue effectively [7,8]. Patient–provider communication is critical, with trust and clear communication significantly influencing adherence decisions. Positive interactions, including clear communication and cultural competency, are strongly associated with improved adherence rates [9,10,11].

Physicians and pharmacists are integral to educating patients, addressing concerns, and implementing interventions to enhance adherence [12,13]. However, medication adherence is often overlooked during consultations, and healthcare professionals struggle to accurately predict patients’ adherence patterns [14,15]. Tools like the Morisky Medication Adherence Scale (MMAS) and electronic monitoring devices offer valuable insights into adherence patterns, enabling targeted interventions [16]. Healthcare professionals employ various effective strategies to improve medication adherence, including dose simplification, patient education, electronic reminders, and reducing patient cost-sharing. Face-to-face interventions, particularly those delivered directly to patients by pharmacists, have demonstrated the most significant impact. The greatest impact on adherence is reflected in significant improvements in clinical and humanistic outcomes, such as improved disease control, reduced hospitalizations, and enhanced quality of life. However, substantial gaps persist, emphasizing the need for continued advancements. The research indicates that healthcare providers should focus on behavioral strategies, particularly those that build habits, rather than relying solely on cognitive approaches aimed at changing knowledge and beliefs [7,8,17,18,19]. Nevertheless, professionals often encounter challenges, such as patients’ reluctance to discuss non-adherence, limited consultation time, and cultural or language barriers, that hinder effective communication and assessment [15,20,21].

While existing studies on medication adherence often focus on the patient’s perspective, there is a noticeable gap in the research examining healthcare professionals’ views on adherence, the barriers they face, and the tools and interventions they use [22]. In Saudi Arabia, the healthcare system operates across multiple levels where adherence interventions may occur, yet the assessment of these aspects from the perspective of healthcare professionals remain underexplored. One possible reason for this under-research is the predominance of patient-centric studies, which tend to overshadow the critical role healthcare professionals play in promoting adherence [1]. Furthermore, the dynamic nature of healthcare delivery in Saudi Arabia presents unique challenges, including high patient-to-practitioner ratios, variations in clinical training, and limited access to adherence assessment tools, which may hinder the consistent application of adherence management practices [23].

Cultural and religious factors unique to Saudi Arabia and other Muslim-majority countries play a significant role in influencing medication adherence [24,25]. During Ramadan, for example, changes in meal and medication schedules may lead to non-adherence, requiring tailored interventions by healthcare providers [26,27]. Additionally, healthcare professionals may face challenges in addressing cultural sensitivities while discussing adherence, which further highlights the importance of culturally specific training and resources [28,29]. Understanding these dimensions is crucial to developing culturally relevant and contextually appropriate adherence interventions tailored to the Saudi healthcare system.

The consequences of this gap are significant. Without understanding healthcare professionals’ perspectives, it becomes challenging to design effective interventions that align with their needs and practical constraints [30,31,32]. This lack of insight may lead to suboptimal adherence strategies, further exacerbating medication non-adherence and its associated clinical and economic burdens. Addressing this research gap also aligns with national efforts under Saudi Vision 2030, which focus on enhancing medication adherence through patient support programs, updated national medicines policies, and medication therapy management services in community pharmacies. Additionally, digital health solutions, such as mobile health applications, are being explored to support adherence [33,34,35,36].

Given the substantial impact of non-adherence on health outcomes and healthcare costs, this study aims to address critical gaps in understanding how physicians and pharmacists in Saudi Arabia manage medication adherence by investigating the methods employed for adherence assessment, the interventions implemented to enhance adherence, and the challenges encountered in practice.

## 2. Materials and Methods

### 2.1. Study Design

The research utilized an online cross-sectional survey conducted over a 9-month period (December 2023 to September 2024). The study aimed to investigate the self-reported behaviors of healthcare professionals in Saudi Arabia regarding their management of patient adherence to prescribed medications.

### 2.2. Population

The study population comprised actively licensed healthcare professionals in Saudi Arabia, specifically targeting physicians and pharmacists directly involved in patient care within healthcare settings. The inclusion criteria required participants to be currently practicing in Saudi Arabia, licensed to work as a physician or pharmacist, and willing to contribute to the research. The exclusion criteria included healthcare professionals who were not involved in direct patient care, were not currently practicing, or were based outside Saudi Arabia to maintain the study’s focus on the local healthcare context.

### 2.3. Sample Size

A sample size of 383 healthcare providers was determined using an online sample size software (Raosoft version 2004 Seattle USA) with a 5% margin of error, 95% confidence interval, 50% response distribution and an estimated population size of 100,000 physicians and pharmacists registered to practice in Saudi Arabia.

### 2.4. Survey Development

The questionnaire was developed based on a thorough review of existing research studies that explored healthcare professionals’ behaviors related to patient medication adherence [37,38,39,40,41]. The survey questions were carefully aligned with the predetermined research objectives and were reviewed by the research team and two clinical pharmacists to ensure relevance and clarity.

The questionnaire comprised 38 items divided into three sections, along with demographic characteristics. The first section assessed the frequency of tools and methods used by healthcare professionals to evaluate patients´ medication adherence, using a 5-point Likert scale ranging from 1 (Never) to 5 (Always). The second section measured the frequency of interventions utilized to enhance patient adherence, also using a 5-point Likert scale from 1 (Never) to 5 (Always). The third section assessed healthcare professionals’ agreement with a list of barriers hindering their ability to assess patient medication adherence, using a 5-point Likert scale from 1 (Strongly Disagree) to 5 (Strongly Agree).

Before launching the full-scale study, the online survey was piloted among a diverse group of 12 healthcare professionals from various regions in Saudi Arabia. This pilot test was conducted to ensure the technical functionality of the survey, identify any formatting errors, and assess the comprehensibility of the questions. The feedback from the pilot participants was used to make minor adjustments, ensuring that the survey was optimized for the main data collection phase.

### 2.5. Data Collection

Physicians and pharmacists from Saudi Arabia were recruited using convenience sampling and invited to participate in the study through various platforms. The invitation provided detailed information about the survey and included a web link to access the questionnaire. Eligibility questions were added at the beginning of the questionnaire to ensure that only actively licensed physicians and pharmacists practicing in Saudi Arabia were able to proceed.

The data collection was conducted using SurveyMonkey, an online survey platform. The questionnaire was distributed electronically via platforms such as WhatsApp, Telegram, and the X application. Additionally, printed copies of the questionnaire, accompanied by a QR code for easy access, were distributed in various healthcare facilities, including hospitals and pharmacies. The questionnaire was also shared through professional networks to ensure a wide reach.

### 2.6. Data Analysis

The collected data were analyzed using Microsoft Excel and Stata (version 16). The survey was designed with forced questions, eliminating the possibility of missing data during the analysis. Likert scale responses were analyzed using the weighted average method, assigning numerical weights to each response to quantify agreement or disagreement, with the results visually represented through stacked bar charts for intuitive interpretation.

To determine if there were statistically significant differences between the responses of physicians and pharmacists, *t*-tests were conducted on weighted averages for each survey item, with assumptions checked beforehand. The normality (Shapiro–Wilk test) and homogeneity of variances (Bartlett’s test) were confirmed. Chi-square tests were used to compare categorical response distributions, ensuring that the assumptions were met. A *p*-value threshold of <0.05 was used to determine statistical significance.

Multivariable regression analyses were conducted to identify the factors associated with adherence assessment methods, interventions to enhance medication adherence, and barriers to adherence assessment. For each domain (methods, interventions, and barriers), a separate linear regression model was constructed using the mean scores of the respective domain as dependent variables. The predictors included demographic and professional characteristics such as gender, years of experience, type of organization, practice setting, and consultation time spent with patients. Each model was evaluated for statistical significance, with a *p*-value threshold of <0.05 considered as significant. Additionally, individual items within each category were analyzed using multivariable regression to explore specific associations while adjusting for other predictors. The results are reported as beta coefficients (β) with corresponding *p*-values and 95% confidence intervals. 

Reliability and internal consistency were assessed using Cronbach’s Alpha for the three main sections: Adherence Assessment Methods, Adherence-Enhancing Interventions, and Barriers to Adherence Assessment. Pearson’s correlation coefficients evaluated the relationships between the items and overall section scores, ensuring the alignment with psychometric evaluation standards.

### 2.7. Ethical Considerations

The ethical approval for this study was granted by the ethical committee of Taif University, Application No. 45-076 on 23 November 2023. Informed consent was obtained from all participants, ensuring their voluntary participation. The survey did not collect any personally identifiable information, and the data were only accessible to the research team.

## 3. Results

A total of 397 individuals participated in the survey. Among these respondents, 322 (81.11%) were pharmacists, and 75 (18.89%) were physicians. The demographic profile showed that 78.34% of the participants were male. About 55.42% of the participants were non-Saudi nationals, and 44.84% were from the Western region of Saudi Arabia. Regarding professional experience, 30.23% of participants had been in practice for 6–10 years, while 26.20% had been practicing for 1–5 years. The majority (74.56%) were employed in private organizations, with 25.44% working in government sectors. In terms of workplace settings, 65.49% of participants were based in community pharmacies, 28.21% in hospitals, and 4.03% in primary care facilities. Concerning patient interactions, approximately 58.70% (233 participants) reported spending only 1–5 minutes discussing medication use with their patients (Table 1).

### 3.1. Utilization of Various Methods for Assessing Patient Medication Adherence

**Figure 1:**
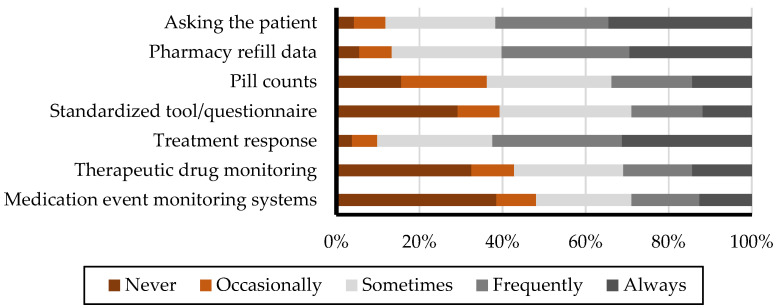
Utilization frequencies of methods and tools for assessing patient medication adherence.

Figure 1 illustrates the frequency of various methods and tools used to assess patient medication adherence. The methods "Treatment response" and “Asking the patient" are the most frequently utilized, as evidenced by their substantial representation in the "Always" and "Frequently" categories. Conversely, methods such as “Standardized tools/questionnaires”, “Therapeutic drug monitoring” and “Medication event monitoring systems (MEMSs)” are employed less often, with a greater proportion of responses falling into the "Never" and "Occasionally" categories, indicating the limited adoption of these methods in practice. 

Table 2 highlights the differences in the methods employed by physicians and pharmacists for evaluating medication adherence. The data reveal significant differences between physicians and pharmacists in several areas. Both groups rely on methods such as treatment response and direct patient questioning, with physicians more frequently assessing adherence through treatment response (weighted average: 4.39 vs. 3.66, *p* < 0.01) and direct questioning (4.28 vs. 3.69, *p* < 0.01) compared to pharmacists. Conversely, pharmacists primarily use pharmacy refill data as their top method (3.81 vs. 3.25, *p* < 0.01), followed by direct questioning (3.69) and treatment response (3.66). Standardized tools or questionnaires (2.95 vs. 1.76, *p* < 0.01) are utilized more frequently by pharmacists than physicians but rank lower compared to other methods for pharmacists. Pill counts are also employed more frequently by pharmacists (3.11 vs. 2.31, *p* < 0.01). Comprehensive data, including weighted averages and statistical comparisons, can be found in the Supplementary File (Table S1).

### 3.2. Utilization of Various Interventions and Strategies to Enhance Medication Adherence

**Figure 2:**
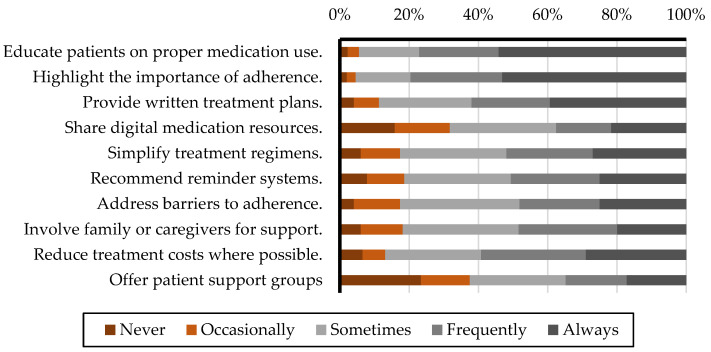
Utilization frequencies of various interventions and strategies to enhance patient medication adherence.

Figure 2 and Table 3 present an overview of the frequency with which healthcare professionals employ various interventions and strategies to enhance patient medication adherence. Both groups prioritize patient education as a key strategy, with physicians educating patients about proper medication use (weighted average: 4.44 vs. 4.19, *p* < 0.01) and highlighting the importance of adherence (4.51 vs. 4.2, *p* < 0.01) more frequently. However, providing written treatment plans is more common among pharmacists (3.96 vs. 3.43, *p* < 0.01). Yet, the use of digital materials is significantly lower among both groups, with pharmacists utilizing them more frequently than physicians (3.26 vs. 2.49, *p* < 0.01). Pharmacists reported recommending reminder systems (3.61 vs. 2.99, *p* < 0.01) and addressing barriers to adherence, including reducing treatment costs (3.75 vs. 3.39, *p* < 0.01), more frequently than physicians. Conversely, physicians slightly favored simplifying treatment regimens (3.57 vs. 3.47, *p* = 0.12) and involving family or caregivers for support (3.47 vs. 3.43, *p* = 0.53), though these differences were not statistically significant. The comprehensive data on the frequency and weighted averages of adherence-enhancing interventions employed by physicians and pharmacists can be found in the Supplementary File (Table S2).

### 3.3. Perceived Barriers to Assessing Patient Medication Adherence

**Figure 3:**
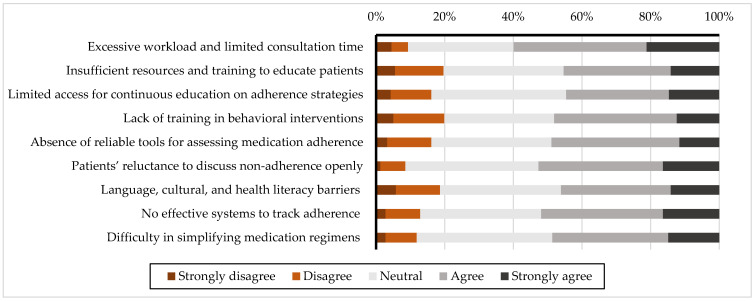
Perceived barriers to assessing patient medication adherence.

Figure 3 and Table 4 highlight the barriers healthcare professionals face in assessing medication adherence. The most significant barrier overall, and specifically for pharmacists, was "Excessive workload and limited consultation time," with approximately 60% of participants rating this barrier as "agree" or "strongly agree" (weighted averages of 3.71 for pharmacists and 3.49 for physicians, *p* < 0.01). Among physicians, however, the "Lack of effective systems to track adherence" emerged as a more prominent concern, with a weighted average of 3.69 compared to 3.49 for pharmacists (*p* < 0.01). Systemic barriers, including the "Absence of reliable tools for assessing medication adherence" and "Language, cultural, and health literacy barriers," were rated higher by pharmacists (3.44 and 3.38, respectively) compared to physicians (3.28 and 3.25, respectively; both *p* < 0.01). Finally, "Difficulty simplifying medication regimens" was rated slightly higher by physicians (3.57) compared to pharmacists (3.47), while communication challenges, such as "Patients’ reluctance to discuss non-adherence openly," were rated slightly higher by pharmacists (3.63) compared to physicians (3.44). However, neither difference was statistically significant (both *p* = 0.12). A breakdown of the perceived barriers to assessing medication adherence, including the statistical significance and weighted averages, is provided in the Supplementary File (Table S3).

### 3.4. Factors Influencing Assessment Methods, Interventions, and Barriers in Medication Adherence Management

The regression analysis identified significant associations between demographic and professional factors and the mean score of barriers to medication adherence. Specifically, gender was a significant predictor, with males reporting fewer overall barriers compared to females (β = −0.2517, *p* = 0.019). Consultation time was another significant factor; participants with shorter consultation times (less than 1 minute: β = −0.6262, *p* = 0.012) reported higher mean scores of barriers. In contrast, the regression analyses of mean scores for adherence assessment methods and interventions did not yield any significant predictors.

When examining individual items within these domains, the analysis revealed nuanced differences in specific practices and challenges. For adherence assessment methods, early-career professionals (1–5 years of experience) were significantly more likely to ask patients directly about adherence (*p* = 0.015), while hospital settings were negatively associated with this practice (*p* = 0.003). The use of therapeutic drug monitoring (TDM) was notably less common in private organizations (*p* = 0.030).

For adherence-enhancing interventions, the analysis showed that simplifying treatment regimens was significantly less common in private organizations (*p* = 0.045). Providing digital materials about medications was positively associated with spending more consultation time with patients (e.g., more than 15 min; *p* = 0.018). Additionally, interventions aimed at minimizing treatment costs were more frequently reported by those with over 15 years of experience (*p* = 0.017) but were less prevalent in private organizations (*p* = 0.013).

The analysis of the barriers to adherence highlighted further individual challenges. Shorter consultation times (e.g., 1–5 minutes) were associated with significantly higher reports of insufficient resources (*p* = 0.027) and a lack of reliable tools for assessing adherence (*p* = 0.013). Additionally, shorter consultation times (e.g., less than 1 min) were significantly linked to challenges in simplifying medication regimens (*p* = 0.025), establishing effective tracking systems (*p* = 0.008), and accessing training opportunities (*p* = 0.021).

### 3.5. Internal Consistency and Correlation Analysis of Survey Measures

The survey demonstrated a strong internal consistency across all sections. Cronbach’s Alpha values were 0.7890 for the Adherence Assessment Methods section, 0.8825 for the Adherence-Enhancing Interventions section, and 0.8379 for the Barriers to Adherence Assessment section, indicating good to excellent reliability. Items within each section showed moderate to strong correlations with their respective total scores, supporting their contribution to the construct being measured. 

Higher scores for the Adherence Assessment Methods section were positively correlated with higher scores for the Adherence-Enhancing Interventions section (r = 0.7035, *p* < 0.001), reflecting the alignment between assessment practices and intervention strategies. The Barriers to Adherence Assessment section showed weak correlations with both Adherence Assessment Methods (r = −0.1014, *p* = 0.0435) and Adherence-Enhancing Interventions (r = −0.0791, *p* = 0.1154), suggesting that the barriers are distinct from practices and interventions.

These findings indicate that the survey instrument is a reliable tool for assessing healthcare professionals’ practices, interventions, and challenges related to medication adherence. The analysis aligns with established psychometric standards, ensuring clarity and robustness in the results.

## 4. Discussion

This study provides a comprehensive assessment of medication adherence management among healthcare professionals in Saudi Arabia, focusing on the methods, interventions, and barriers encountered in clinical practice. The findings reveal critical insights into current practices and potential areas for improvement in medication adherence management.

The results reveal a predominant reliance on direct patient inquiries and treatment response evaluation as the primary methods for assessing adherence. Nearly half of the respondents frequently employed these practical approaches, which are valued for their simplicity and immediacy. However, the underutilization of standardized tools, such as questionnaires and MEMSs, by both physicians and pharmacists suggest a significant gap in adopting structured, evidence-based adherence assessment methods. This limited use may reflect challenges such as resource constraints, lack of training, or insufficient awareness of the benefits of these tools. Additionally, excessive workload and limited consultation time, as identified in this study, may further deter the adoption of these time-intensive tools. Comparatively, studies from Europe report higher utilization rates of standardized tools and MEMSs, likely due to stronger institutional support, advanced healthcare infrastructure, and comprehensive professional training [37,38]. Encouraging the integration of validated tools in Saudi Arabia could enhance the accuracy and consistency of adherence assessments.

The findings also highlight patient education as the most frequently employed intervention by both physicians and pharmacists, underscoring its universal recognition as a cornerstone of adherence improvement. Traditional methods, such as one-on-one education and providing written treatment plans, were preferred over digital resources and support groups. This preference indicates a reliance on personalized and direct communication. However, the limited use of digital tools, reminder systems, and support groups reveals untapped opportunities to incorporate technology into adherence interventions. Non-technological approaches, such as patient education and written treatment plans, are common in both Saudi Arabia and Europe [37,38]. In contrast, Australian healthcare professionals, particularly pharmacists, emphasize practical tools like dose administration aids and computerized prescription monitoring, which remain underutilized in Saudi Arabia [42]. These differences likely reflect variations in healthcare infrastructure and cultural attitudes toward medication management.

The observed differences between physicians and pharmacists in their adherence assessment and intervention strategies reflect their distinct roles and responsibilities within the healthcare system. Pharmacists’ preference for written treatment plans and pharmacy refill data aligns with their primary role in dispensing medications and tracking refill histories. Conversely, physicians rely more frequently on verbal patient inquiry and treatment response due to their focus on diagnosing and monitoring clinical outcomes. Time constraints during consultations may further drive physicians’ reliance on verbal communication, as it offers a quicker way to gather adherence information compared to reviewing records or using structured tools [42].

Our study highlights that pharmacists are more likely to address financial and logistical barriers, such as reducing treatment costs and resolving patient concerns, compared to physicians. These findings underscore the critical role of pharmacists in bridging gaps in medication adherence, particularly in community pharmacy settings where they directly interact with patients [43]. The underutilization of digital resources, despite their potential to enhance patient engagement and adherence tracking, points to a need for targeted training and investment in technology to modernize adherence strategies.

The barriers identified in this study resonate with global challenges in adherence management, such as excessive workload, limited consultation time, and inadequate tracking systems [44,45]. This finding highlights the global nature of these obstacles in managing medication adherence, despite regional differences. These systemic issues, compounded by communication barriers like patient reluctance to discuss non-adherence, reflect the multifaceted nature of adherence challenges. Pharmacists reported more concerns regarding the absence of reliable tools and systemic barriers such as language and health literacy, highlighting the need for tailored solutions to address these gaps.

Interestingly, physicians emphasized the lack of effective systems for tracking adherence, underscoring a broader need for integrated healthcare systems that support adherence monitoring [46,47]. The findings further underscore the importance of cultural and structural factors, such as health literacy and institutional support, which significantly influence the ability of healthcare professionals to manage adherence effectively.

The findings highlight barriers related to language, cultural differences, and health literacy. These barriers are especially relevant in Saudi Arabia, given its diverse population and large number of foreign residents. With more than 40% of the population being non-Saudis as of 2022, healthcare professionals face significant challenges in addressing adherence among patients with diverse linguistic abilities, cultural backgrounds, and health literacy levels [48]. These factors are particularly pronounced among older patients and expatriates, who may require tailored communication strategies and culturally sensitive interventions.

The regression analysis highlights the key factors influencing medication adherence management. Shorter consultation times were strongly linked to greater barriers, including insufficient resources and difficulties in simplifying regimens, highlighting the need for workflow optimization to allow more comprehensive patient interactions. Private organizations demonstrated a lower adoption of therapeutic drug monitoring and regimen simplification, pointing to resource limitations and the need for targeted interventions. Longer consultation times were associated with a greater use of digital materials for patient education, emphasizing the value of extended interactions in enhancing adherence strategies. These findings stress the importance of institutional support, resource allocation, and tailored training to address systemic barriers and optimize adherence practices.

The findings of this study have important implications for both healthcare practice and policymaking in Saudi Arabia and offer insights relevant to other global contexts, highlighting the need to address regional challenges in medication adherence management. While there are commonalities in approaches across different regions, notable differences, particularly in the use of standardized tools and technological interventions, emphasize the importance of tailored, region-specific strategies. Compared to the broader evidence base, these findings highlight that healthcare professionals can benefit from a balanced approach, integrating traditional methods with evidence-based tools and innovative solutions. Policymakers should prioritize the development of national guidelines that standardize adherence practices, provide institutional support, and promote the integration of technology in adherence monitoring and interventions. Additionally, addressing systemic barriers, such as workload constraints and limited consultation time, requires organizational changes, such as optimizing healthcare workflows and allocating adequate time for patient counseling. Investment in continuous professional development programs focused on adherence strategies, behavioral interventions, and cultural competence will further empower healthcare professionals to navigate adherence challenges effectively [49]. Additionally, enhancing interprofessional collaboration, particularly between physicians and pharmacists, could improve adherence management by leveraging each profession’s strengths. Pharmacists have been shown to have a significant impact on medication adherence through direct patient interactions and counseling [17,18,50].

In-depth qualitative research should be prioritized to gain a deeper understanding of the barriers healthcare professionals face in assessing and managing medication adherence. These studies can provide rich, contextual insights into challenges like cultural and religious influences, systemic limitations, and communication barriers. Longitudinal studies are also essential to track trends in adherence practices and assess the sustained impact of interventions over time. These studies could evaluate how systemic changes, such as policy reforms under Saudi Vision 2030, influence healthcare professionals’ approaches to medication adherence. Additionally, interventional studies, such as randomized controlled trials tailored to the Saudi healthcare system, should investigate the effectiveness of culturally adapted strategies. These could include digital tools, reminder systems, and educational interventions designed to address specific adherence challenges and improve patient outcomes. Addressing the identified barriers requires targeted initiatives, such as the implementation of robust adherence tracking systems and comprehensive training programs. Overall, future efforts should translate research findings into practice by implementing evidence-based guidelines and best practices, ultimately aiming to enhance medication adherence and improve patient outcomes.

This study possesses several strengths, notably being the first of its kind in Saudi Arabia to focus on medication management practices among healthcare professionals themselves rather than solely on patient perspectives. Additionally, it offers valuable insights into the variability of beliefs and behaviors across different regions of Saudi Arabia, shedding light on potential regional disparities in medication adherence management. Despite challenges in participant recruitment, including difficulties in certain regions and professions, the study successfully engaged healthcare professionals from diverse work settings, regions, and demographic backgrounds, providing a comprehensive understanding of medication adherence practices. However, several limitations should be acknowledged. The use of convenience sampling may limit the generalizability of the findings, particularly as participant recruitment fell short of the desired sample size in certain subgroups, particularly among physicians and in specific regions. This imbalance may limit the generalizability of findings and introduce regional and professional biases [51]. Furthermore, due to the nature of convenience sampling, it was not possible to calculate a response rate, as there was no fixed sampling frame or list from which participants were invited. Finally, the voluntary nature of participation and the use of social media platforms for survey distribution raise concerns about selection bias, potentially favoring healthcare professionals with greater digital literacy, active engagement in online professional networks, or a stronger interest in medication management, which may overestimate the proportion utilizing adherence-enhancing interventions compared to the broader healthcare professional population [52].

## 5. Conclusions

This study highlights key differences in medication adherence management between physicians and pharmacists in Saudi Arabia, reflecting their distinct roles. Physicians rely on direct inquiries and treatment response evaluation, while pharmacists frequently use pharmacy refill data. Barriers such as excessive workload, limited consultation time, and inadequate tracking systems hinder the adoption of evidence-based tools like MEMSs and standardized questionnaires. To improve adherence management, interventions should be role-specific: streamlined tracking systems for physicians and enhanced counseling support for pharmacists. Collaborative efforts between the two groups can maximize the impact of adherence strategies. Future research should explore cultural and systemic barriers through qualitative studies and evaluate innovative, culturally adapted interventions in the Saudi context. Policymakers must address systemic challenges, optimize workflows, and provide institutional support to improve adherence practices and patient outcomes.

## Figures and Tables

**Table 1:** Demographic characteristics of study participants (N = 397).

Characteristic		N (%)/Mean ± SD
Profession		
	Physicians	75 (18.89)
	Pharmacists	322 (81.11)
Gender		
	Male	311 (78.34)
	Female	86 (21.66)
Nationality		
	Saudi	177 (44.58)
	Non-Saudi	220 (55.42)
Age (in years)		34.88 ± 8.37
Province Region		
	Central	79 (19.90)
	Eastern	56 (14.11)
	Northern	29 (7.30)
	Southern	55 (13.85)
	Western	178 (44.84)
Years Since Qualifying		
	Less than 1 year	20 (5.04)
	1–5 years	104 (26.20)
	6–10 years	120 (30.23)
	11–15 years	82 (20.65)
	Over 15 years	71 (17.99)
Type of Healthcare Organization		
	Private	296 (74.56)
	Government	101 (25.44)
Work Settings		
	Hospital	112 (28.21)
	Primary care	16 (4.03)
	Community pharmacy	260 (65.49)
	Other	9 (2.27)
Length of Time Spent Talking to Patients About Their Use of Medications.		
	No time at all	3 (0.8)
	Less than 1 min	29 (7.3)
	1–5 min	233 (58.7)
	6–10 min	93 (23.4)
	11–15 min	17 (4.3)
	More than 15 min	22 (5.5)

**Table 2:** Weighted average frequency of methods used by healthcare professionals to assess medication adherence.

	Pharmacists	Physicians	*p*-Value (*t*-test)
Asking the Patient	3.69	4.28	<0.01
Pharmacy Refill Data	3.81	3.25	<0.01
Pill Counts	3.11	2.31	<0.01
Standardized Tool/Questionnaire	2.95	1.76	<0.01
Treatment Response	3.66	4.39	<0.01
Therapeutic Drug Monitoring	2.70	2.71	0.88
Medication Event Monitoring Systems	2.68	2.0	<0.01

**Table 3:** Weighted average frequency of various interventions and strategies to enhance medication adherence.

	Pharmacists	Physicians	*p*-Value (*t*-test)
Educate Patients on Proper Medication Use	4.19	4.44	<0.01
Highlight the Importance of Adherence	4.2	4.51	<0.01
Provide Written Treatment Plans	3.96	3.43	<0.01
Share Digital Medication Resources	3.26	2.49	<0.01
Simplify Treatment Regimens	3.47	3.57	0.12
Recommend Reminder Systems	3.61	2.99	<0.01
Address Barriers to Adherence	3.55	3.36	<0.01
Involve Family or Caregivers for Support	3.43	3.47	0.53
Reduce Treatment Costs Where Possible	3.75	3.39	<0.01
Offer Patient Support Groups	3.01	2.47	<0.01

**Table 4:** Weighted average of perceived barriers to assessing patient medication adherence.

	Pharmacists	Physicians	*p*-Value (*t*-test)
Excessive Workload and Limited Consultation Time	3.71	3.49	<0.01
Insufficient Resources and Training to Educate Patients	3.34	3.33	<0.01
Limited Access for Continuous Education on Adherence Strategies	3.41	3.28	0.002
Lack of Training in Behavioral Interventions	3.38	3.27	<0.01
Absence of Reliable Tools for Assessing Medication Adherence	3.44	3.28	<0.01
Patients’ Reluctance to Discuss Non-adherence Openly	3.63	3.44	0.12
Language, Cultural, and Health Literacy Barriers	3.38	3.25	<0.01
No Effective Systems to Track Adherence	3.49	3.69	<0.01
Difficulty in Simplifying Medication Regimens	3.47	3.57	0.12

## Data Availability

The datasets used and analyzed during the current study are available from the corresponding author upon reasonable request.

## Supplementary Materials

The following supporting information can be downloaded at: https://www.mdpi.com/article/10.3390/healthcare13030347/s1, Table S1: The frequency and statistical analysis of methods used for assessing patient medication adherence; Table S2: The frequency and statistical analysis of adherence-enhancing interventions used by healthcare professionals; Table S3: The perceived barriers to assessing patient medication adherence among healthcare professionals.
