# Supplementary material for: A Cross-Sectional Survey on the Management of Medication Adherence Among Healthcare Professionals in Saudi Arabia
Source: Healthcare (Basel). 2025 Feb 6;13(3):347. doi: 10.3390/healthcare13030347 (PMC11817170; doi:10.3390/healthcare13030347)
Supplement: Supplementary file 1 [file healthcare-13-00347-s001.zip › healthcare-3396438-supplementary.pdf]

Table S1: How often do you use the following methods or tools for assessing patient medication adherence?

Question	Group	Never	Occasionally	Sometimes	Frequently	Always	Weighted Average	P Value Chi Square	P Value t test
1. Asking the patient e.g., do you miss any dose, did you complete your doses	Physicians	1 (1.33%)	5 (6.67%)	8 (10.67%)	19 (25.33%)	42 (56.00%)	4.28	<0.001	<0.001
	Pharmacists	16 (4.97%)	25 (7.76%)	97 (30.12%)	89 (27.64%)	95 (29.50%)	3.69		
2. Pharmacy refill data. List of medications filled by a pharmacy for the patient	Physicians	10 (13.33%)	11 (14.67%)	20 (26.67%)	18 (24.00%)	16 (21.33%)	3.25	0.001	<0.001
	Pharmacists	12 (3.73%)	20 (6.21%)	85 (26.40%)	104 (32.30%)	101 (31.37%)	3.81		
3. Pill counts The medication bottle or strips dispensed during the previous visit are brought by the patient.	Physicians	24 (32.00%)	22 (29.33%)	16 (21.33%)	8 (10.67%)	5 (6.67%)	2.31	<0.001	<0.001
	Pharmacists	38 (11.80%)	60 (18.63%)	103 (31.99%)	69 (21.43%)	52 (16.15%)	3.11		
4. Standardized tool/questionnaire e.g., Morisky Medication Adherence Scale (MMAS) and the Medication Adherence Report Scale (MARS)	Physicians	49 (65.33%)	8 (10.67%)	7 (9.33%)	9 (12.00%)	2 (2.67%)	1.76	<0.001	<0.001
	Pharmacists	67 (20.81%)	32 (9.94%)	119 (36.96%)	59 (18.32%)	45 (13.98%)	2.95		
5. Treatment response e.g., sign and symptoms improvement.	Physicians	2 (2.67%)	1 (1.33%)	5 (6.67%)	25 (33.33%)	42 (56.00%)	4.39	<0.001	<0.001
	Pharmacists	13 (4.04%)	23 (7.14%)	105 (32.61%)	99 (30.75%)	82 (25.47%)	3.66		
6. Therapeutic Drug Monitoring (TDM) Measures medication concentration in patient blood.	Physicians	21 (28.00%)	14 (18.67%)	16 (21.33%)	14 (18.67%)	10 (13.33%)	2.71	0.094	0.876
	Pharmacists	108 (33.54%)	27 (8.39%)	88 (27.33%)	52 (16.15%)	47 (14.60%)	2.7		
7. Medication event monitoring systems (MEMSs) Special bottle caps that record each time the bottle is opened	Physicians	44 (58.67%)	6 (8.00%)	11 (14.67%)	9 (12.00%)	5 (6.67%)	2	0.003	<0.001
	Pharmacists	109 (33.85%)	32 (9.94%)	80 (24.84%)	56 (17.39%)	45 (13.98%)	2.68		

Table S2: How often do you use the following adherence enhancing interventions with your patients?

Question	Group	Never	Occasionally	Sometimes	Frequently	Always	Weighted Average	P Value Chi Square	P Value t test
**1. Educating the patient about the appropriate use of their medications.**	Physicians	2 (2.67%)	2 (2.67%)	4 (5.33%)	20 (26.67%)	47 (62.67%)	4.44	0.046	<0.001
	Pharmacists	7 (2.17%)	11 (3.42%)	65 (20.19%)	71 (22.05%)	168 (52.17%)	4.19		
**2. Educating the patient about the importance of adherence**	Physicians	1 (1.33%)	2 (2.67%)	3 (4.00%)	21 (28.00%)	48 (64.00%)	4.51	0.032	<0.001
	Pharmacists	7 (2.17%)	8 (2.48%)	60 (18.63%)	84 (26.09%)	163 (50.62%)	4.2		
**3. Providing patients with written information about their treatment plan.**	Physicians	6 (8.00%)	10 (13.33%)	25 (33.33%)	14 (18.67%)	20 (26.67%)	3.43	0.007	<0.001
	Pharmacists	10 (3.11%)	19 (5.90%)	81 (25.16%)	76 (23.60%)	136 (42.24%)	3.96		
**4. Providing patients with digital materials about their medication.**	Physicians	21 (28.00%)	19 (25.33%)	18 (24.00%)	11 (14.67%)	6 (8.00%)	2.49	<0.001	<0.001
	Pharmacists	42 (13.04%)	44 (13.66%)	104 (32.30%)	52 (16.15%)	80 (24.84%)	3.26		
**5. Simplifying the treatment regimen.**	Physicians	6 (8.00%)	9 (12.00%)	20 (26.67%)	24 (32.00%)	16 (21.33%)	3.47	0.399	0.122
	Pharmacists	18 (5.59%)	36 (11.18%)	102 (31.68%)	75 (23.29%)	91 (28.26%)	3.57		
**6. Advising patient to use reminder Systems**	Physicians	11 (14.67%)	13 (17.33%)	27 (36.00%)	14 (18.67%)	10 (13.33%)	2.99	0.002	<0.001
	Pharmacists	20 (6.21%)	30 (9.32%)	95 (29.50%)	88 (27.33%)	89 (27.64%)	3.61		
**7. Addressing any concerns or barriers**	Physicians	5 (6.67%)	14 (18.67%)	22 (29.33%)	17 (22.67%)	17 (22.67%)	3.36	0.346	0.004
	Pharmacists	11 (3.42%)	39 (12.11%)	115 (35.71%)	75 (23.29%)	82 (25.47%)	3.55		
**8. Engage family members or caregivers**	Physicians	6 (8.00%)	7 (9.33%)	24 (32.00%)	22 (29.33%)	16 (21.33%)	3.47	0.853	0.534
	Pharmacists	18 (5.59%)	41 (12.73%)	109 (33.85%)	91 (28.26%)	63 (19.57%)	3.43		
**9. Trying to Minimize treatment costs**	Physicians	10 (13.33%)	7 (9.33%)	17 (22.67%)	26 (34.67%)	15 (20.00%)	3.39	0.02	<0.001
	Pharmacists	16 (4.97%)	19 (5.90%)	93 (28.88%)	94 (29.19%)	100 (31.06%)	3.75		
**10. Offering support groups for patients.**	Physicians	23 (30.67%)	17 (22.67%)	18 (24.00%)	11 (14.67%)	6 (8.00%)	2.47	0.015	<0.001
	Pharmacists	70 (21.74%)	39 (12.11%)	92 (28.57%)	59 (18.32%)	62 (19.25%)	3.01		

Table S3: Please indicate the extent to which you agree with each of the following barriers that hinders your ability to assess patient medication adherence:

Question	Group	Strongly Disagree	Disagree	Neutral	Agree	Strongly Agree	Weighted Average	P Value Chi Square	P Value t test
**1. Excessive workload/short consultation times.**	Physicians	7 (9.33%)	6 (8.00%)	20 (26.67%)	27 (36.00%)	15 (20.00%)	3.49	0.115	<0.001
	Pharmacists	11 (3.42%)	13 (4.04%)	102 (31.68%)	127 (39.44%)	69 (21.43%)	3.71		
**2. Insufficient resources and training to educate patients effectively**	Physicians	8 (10.67%)	8 (10.67%)	22 (29.33%)	25 (33.33%)	12 (16.00%)	3.33	0.173	<0.001
	Pharmacists	14 (4.35%)	48 (14.91%)	117 (36.34%)	99 (30.75%)	44 (13.66%)	3.34		
**3. Lack of opportunities for continuous education**	Physicians	7 (9.33%)	7 (9.33%)	27 (36.00%)	26 (34.67%)	8 (10.67%)	3.28	0.095	0.002
	Pharmacists	10 (3.11%)	40 (12.42%)	129 (40.06%)	93 (28.88%)	50 (15.53%)	3.41		
**4. Lack of training in behavioural interventions**	Physicians	6 (8.00%)	11 (14.67%)	21 (28.00%)	31 (41.33%)	6 (8.00%)	3.27	0.369	<0.001
	Pharmacists	14 (4.35%)	48 (14.91%)	106 (32.92%)	111 (34.47%)	43 (13.35%)	3.38		
**5. Lack of reliable and practical tools**	Physicians	4 (5.33%)	11 (14.67%)	26 (34.67%)	28 (37.33%)	6 (8.00%)	3.28	0.651	<0.001
	Pharmacists	9 (2.80%)	40 (12.42%)	113 (35.09%)	120 (37.27%)	40 (12.42%)	3.44		
**6. Patients' reluctance to openly discuss non-adherence**	Physicians	3 (4.00%)	12 (16.00%)	20 (26.67%)	29 (38.67%)	11 (14.67%)	3.44	0.001	0.122
	Pharmacists	2 (0.62%)	17 (5.28%)	134 (41.61%)	115 (35.71%)	54 (16.77%)	3.63		
**7. Language barriers, cultural differences, and health literacy issues**	Physicians	10 (13.33%)	8 (10.67%)	21 (28.00%)	25 (33.33%)	11 (14.67%)	3.25	0.028	<0.001
	Pharmacists	13 (4.04%)	43 (13.35%)	119 (36.96%)	102 (31.68%)	45 (13.98%)	3.38		
**8. Absence of effective tracking systems**	Physicians	3 (4.00%)	3 (4.00%)	21 (28.00%)	35 (46.67%)	13 (17.33%)	3.69	0.073	<0.001
	Pharmacists	8 (2.48%)	37 (11.49%)	119 (36.96%)	106 (32.92%)	52 (16.15%)	3.49		
**9. Challenge of simplifying medication regimens**	Physicians	3 (4.00%)	5 (6.67%)	22 (29.33%)	36 (48.00%)	9 (12.00%)	3.57	0.048	0.122
	Pharmacists	8 (2.48%)	31 (9.63%)	135 (41.93%)	98 (30.43%)	50 (15.53%)	3.47		
